# Fovea-UNet: detection and segmentation of lymph node metastases in colorectal cancer with deep learning

**DOI:** 10.1186/s12938-023-01137-4

**Published:** 2023-07-21

**Authors:** Yajiao Liu, Jiang Wang, Chenpeng Wu, Liyun Liu, Zhiyong Zhang, Haitao Yu

**Affiliations:** 1 School of Electrical and Information Engineering, Tianjin University, Tianjin, China; 2 Department of Pathology, Tangshan Gongren Hospital, Tangshan, China

**Keywords:** Medical image segmentation, Colorectal cancer, Fovea in human retina, Adaptive resolution, Feature importance-aware, Attention mechanism, Lightweight backbone network

## Abstract

**Background:**

Colorectal cancer is one of the most serious malignant tumors, and lymph node metastasis (LNM) from colorectal cancer is a major factor for patient management and prognosis. Accurate image detection of LNM is an important task to help clinicians diagnose cancer. Recently, the U-Net architecture based on convolutional neural networks (CNNs) has been widely used to segment image to accomplish more precise cancer diagnosis. However, the accurate segmentation of important regions with high diagnostic value is still a great challenge due to the insufficient capability of CNN and codec structure in aggregating the detailed and non-local contextual information. In this work, we propose a high performance and low computation solution.

**Methods:**

Inspired by the working principle of Fovea in visual neuroscience, a novel network framework based on U-Net for cancer segmentation named Fovea-UNet is proposed to adaptively adjust the resolution according to the importance-aware of information and selectively focuses on the region most relevant to colorectal LNM. Specifically, we design an effective adaptively optimized pooling operation called Fovea Pooling (FP), which dynamically aggregate the detailed and non-local contextual information according to the pixel-level feature importance. In addition, the improved lightweight backbone network based on GhostNet is adopted to reduce the computational cost caused by FP.

**Results:**

Experimental results show that our proposed framework can achieve higher performance than other state-of-the-art segmentation networks with 79.38% IoU, 88.51% DSC, 92.82% sensitivity and 84.57% precision on the LNM dataset, and the parameter amount is reduced to 23.23 MB.

**Conclusions:**

The proposed framework can provide a valid tool for cancer diagnosis, especially for LNM of colorectal cancer.

## Background

Colorectal cancer (CRC) is the third most common cancer and the third leading cause of cancer death in the world [1]. In percentage terms, CRC accounts for 10% of the worldwide cancer incidence and 9–10% of the global cancer deaths [2]. Lymph node metastasis (LNM) is the main metastasis mode of CRC. Accurate diagnosis of LNM provides a solid foundation for the subsequent postoperative management and prognostic estimation. Patients diagnosed with LNM should undergo lymph node dissection surrounding the colon region to prevent further spreading. However, the diagnostic results of LNM are usually artificially given by clinicians with reference to medical images, which may cause inaccurate diagnosis when clinicians are under heavy work and long-time fatigue operation. Hence, an automatic and reliable LNM diagnosis is highly demanded for assisting clinicians in the diagnostic process.

In recent years, convolutional neural networks (CNNs) have shown great potential in the field of medicine, and more specifically in diagnostic medicine, initial results from the application of deep learning to metastasis diagnosis are very promising [3, 4]. Within CNNs, architectures inspired from the U-Net [5] have been widely used for medical segmentation due to their unique ability to analyze features with an encoder–decoder structure [6–8]. They can leverage an end-to-end training paradigm with input images. This makes it possible to segment LNM region and provide a consistent interpretation of the results [9]. To enhance the feature expression abilities of medical image, researchers proposed multiple ways including the introduction of multi-model combination, multi-branching, and attention mechanism. U-Net ++ [10] integrates U-Net structures of different sizes into a network. The encoder and decoder subnetworks perform feature fusion through a series of nested, dense skip connections to reduce the semantic loss between the feature mappings. Double U-Net [11] stacks two U-Net architectures on top of each other. The additional U-Net network is adopted to learn high-level global features, and then these features are fused with the results from the original U-Net in the final decoder. Triple U-Net [12] includes an RGB branch, a HE branch and a segmentation branch. The features extracted from RGB and HE branches are fused to the segmentation branch to learn better representations. Attention U-Net [13] enables the model to utilize the detailed information of features and enhance the mapping and expression of features, by adding a mechanism of attention gates (Ags) to the encoder and decoder. Ags implicitly generate soft region suggestions, highlighting salient features useful for specific tasks. The abovementioned studies improve the structure of U-Net and achieve good results in medical image segmentation, it is easy to cause the imbalance of detailed and non-local contextual information extraction due to the inherent limitations of the CNN and codec structure [14–16]. This problem prevents neural networks from effectively learning general patterns of LNM. To overcome this problem, it is necessary to consider the precise boundaries of different LNM regions and explore their contextual dependences, so that LNM regions can be completely segmented from the intricate tissue background. Thus, the key challenge of this problem is how to achieve the aggregation of detailed and non-local contextual information.

**Fig. 1:**
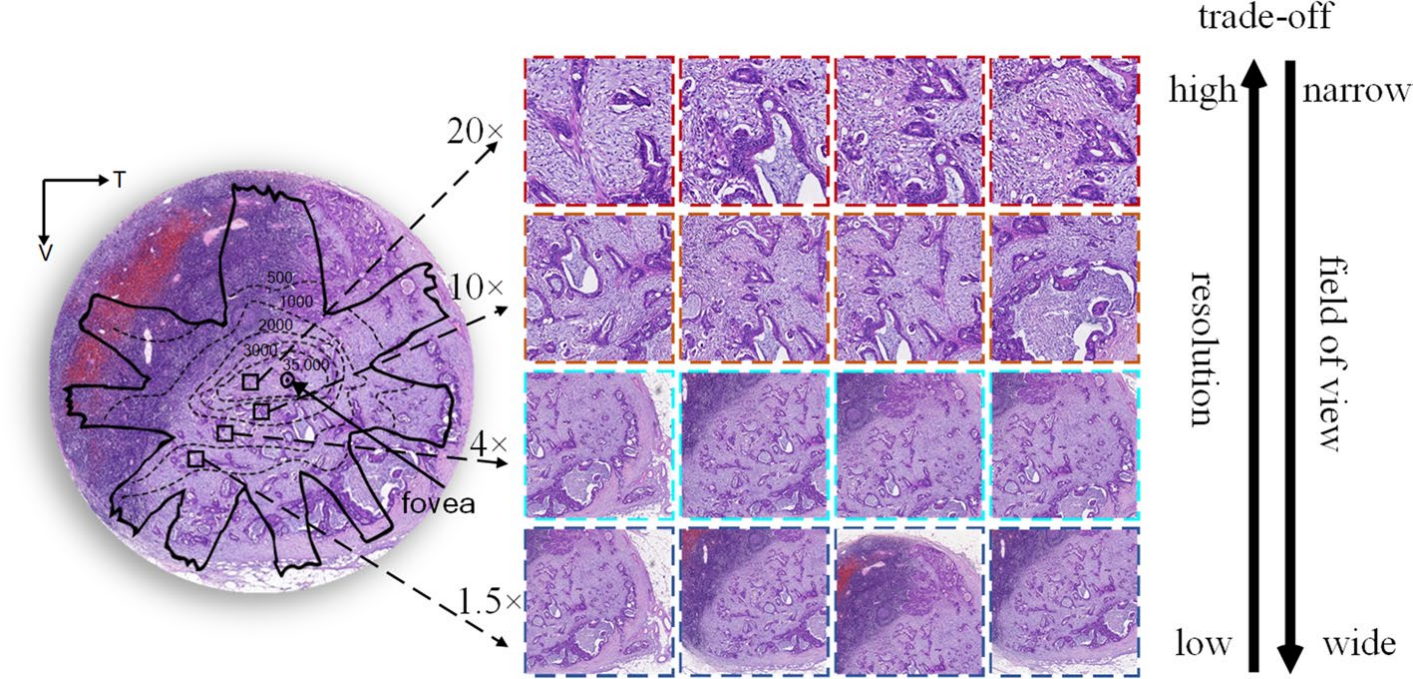
Inspiration of Fovea-UNet. Left, the map shows the LNM images from the perspective of the human eyeball, and the isodensity lines of retinal photoreceptors in the human retina are drawn on the eyeball. Right, examples from different resolutions, correspond to portions with different photoreceptor densities

In the visual neuroscience, the aggregation process belongs to a high visual acuity system, where the retinal fovea contributes to resolving fine spatial detail and the other portion of the retina receive a blurred but wide range field of view [17–19]. For example, in Fig. 1, the distribution of retinal photoreceptor cells on the eyeball is hugely uneven, and that many of them concentrate at the fovea. While in the peripheral portion of the fovea, photoreceptor cells decline rapidly with increasing distance from the fovea. In other words, the fovea has high resolution and the peripheral portion has low resolution. Thus, the fovea can clearly distinguish and recognize the detailed information, and the low-resolution portion surrounding the fovea can obtain the non-local contextual information for quick judgment. Inspired by the fovea of the human visual system, the paper proposes the Fovea-Unet, a lightweight architecture that performs effective LNM segmentation of medical images by devising a Fovea Pooling (FP) method to aggregate the detailed and non-local contextual information in the U-Net encoder. The FP consists of an importance-aware module and the pooling layer with adaptive radius. First, the pixel-level importance of features in the spatial domain is calculated through the importance-aware module that is built on the attention mechanism. Then, the pooling layer aggregates the features with variable pooling radius with an inverse trend of importance. The proposed FP is used in aggregating detailed and non-local contextual information by applying adaptive pooling layers with different radii which handle the segmentation of the region most relevant to LNM at different resolutions. Unlike other U-Net variants adding attention mechanisms, FP overcomes the inherent limitation that the CNN and codec structure cannot balance detailed and non-local contextual information by improving pooling. This operation ensures that the FP can better obtain the non-local contextual information in a full field of view while keeping the reservation of detailed information.

However, the remarkable thing is the importance-aware module of FP will bring the huge computational burden to the entire network. The feasible solution is to reduce the calculation burden via carrying out an efficient and lightweight neural architecture design [20–22]. To this end, we introduce the GhostNet [23] as backbone network for feature extraction, which is a lightweight network that can reduce the calculation cost while retaining the intrinsic features. But directly applying it as the backbone network of LNM segmentation will degrade the segmentation performance because of the intrinsic feature maps calculated by the normal convolution layers may yield insufficient detailed information. Inspired by the theory of neural network representations similarity [24], which has successfully shown to be powerful in providing insights on properties of representations within the neural network, we adopt Hilbert–Schmidt independence criterion (HSIC) to improve the diversity of features. Thus, the improved GhostNet, called nHSIC–GhostNet (H-GhostNet), has the ability to learn the full intrinsic information of the input medical images. Specifically, a similarity constraint, namely, HSIC, within each layer is used as the regularization of the training process and targets to boost the diversity of intrinsic features. Through the HSIC regularization term, the proposed H-GhostNet has the capability of obtaining more feature information and redundancy, which facilitates the accuracy of segmentation results.

In summary, the main contributions of this paper are as follows.

1. We develop an importance-aware Fovea Pooling (FP) to enable the network can focus on the region most relevant to LNM, which is a novel adaptive aggregation pooling method. FP provides a better alternative trade-off that takes both detailed information and non-local contextual information into consideration.
2. We propose an improved H-GhostNet as lightweight backbone network to promote the ability of discriminative and heterogeneous feature extraction through an intrinsic feature-based regularization term. The proposed training strategy cooperates the Ghost convolution layer and HSIC regularization to gain the effective feature representations while maintaining a little amount of computation.
3. We demonstrate the effectiveness of our proposed Fovea-Unet on a practical diagnostic task challenging task. The LNM for colorectal cancer dataset is collected and well-annotated. In addition, comprehensive experiments are conducted and show that our proposed method outperforms state-of-the-art metastasis segmentation methods in the segmentation accuracy and efficiency.

The paper is organized as follows. The LNM segmentation experimental results are given in “Results” section. The discussion based on the experimental results is give in “Discussion” section. This paper is summarized in “Conclusion” section. The proposed Fovea-Unet neural network is given in “Methods” section.

## Results

### Datasets description

In this work, we design the Fovea-Unet to detect colorectal cancer metastasis and segment lymph node metastasis regions. We collected paraffin samples from curative resection of colorectal cancer with lymph node metastasis from Tangshan Gongren Hospital from January 2016 to December 2018. All samples were followed by the process of hematoxylin–eosin (HE) staining, soaked in 10% neutral formalin solution for half an hour to fix the shape of tissues, wrapped into paraffin for half an hour for dehydrating, sectioned on a paraffin microtome, and then dewaxed and stained by HE. Finally, the digital tomography scanner was applied to scan pathological sections into 81 whole slide images (WSIs). The lymph node metastasis regions in WSIs were viewed with K-Viewer software (version 1.5.2.5; KFBIO; http://www.kfbio.cn) at a specific rate, such as × 10 magnification and × 20 magnification, which ensure that the field of view can cover the single metastatic region. In this way, all metastatic regions from the WSIs were manually extracted and resized to 1024*1024 uniformly. After labeling, these metastatic regions with annotations were used to construct the dataset. Table 1 shows the partition of the dataset. Metastatic regions were adopted as input images to achieve high-precision pixel-level segmentation. It should be noted that the collection of these images was approved by the Ethics Committee of Tangshan Gongren Hospital (Grant No. GRYY-LL-2019-50).

**Table 1:** Overview of the training and testing LNM datasets

Datasets	Metastatic WSIs	Metastatic regions
Train	57	451
Test	24	173
Total	81	624

### Experiment settings

The proposed Fovea-Unet is implemented based on the Pytorch 1.8 framework and is trained with one NVIDIA A100 SXM4 GPU with 80 GB RAM. In the model, we use the Adam with parameters beta1 of 0.9 and beta2 of 0.999 to train the whole end-to-end network. The backbone is based on GhostNet pre-trained on ImageNet. In the training stage of 300 iterations, the freeze training method is adopted at the first 30 iterations to put more computing resources on training the network parameters containing the FP modules while preventing the pre-trained weights of backbone network from being destroyed, which can improve the training efficiency. In this stage, the learning rate is set to 10–4. After the freezing stage, all the parameters in the model participate in the training process and the learning rate is set to 10–5, the mini-batch size is set to 4. In each stage of the encoder subnetwork, all feature maps are first reduced to one-quarter of the original number of channels using the prior convolution layer. In the FP, we use the adaptive reflect padding to retain the same size. Besides, data augmentation strategies are utilized to enhance the dataset diversity, and the dataset was randomly divided into the training set and the test set in a ratio of 8:2.

To accurately evaluate the segmentation accuracy, in this paper, we used the intersection over union (*IoU*), dice similarity coefficient (*DSC*), Sensitivity (*Sen*), Specificity (*Sp*), and Precision (*Pre*) as the main evaluation metrics, which are defined below:

1 $$\begin{gathered} IoU = \frac{TP}{{TP + FP + FN}} \hfill \\ DSC = \frac{2 * TP}{{2 * TP + FP + FN}} \hfill \\ Sen = \frac{TP}{{TP + FN}} \hfill \\ Sp = \frac{TN}{{TN + FP}} \hfill \\ Pre = \frac{TP}{{TP + FP}} \hfill \\ \end{gathered}$$

where *FP*, *TN*, *FN*, *TP* denote the number of false positive, true negative, false negative and true positive pixels, respectively. *IoU* ∈ (0, 1) is the ratio between the intersection and union of LNM regions in the ground truth and network segmentation results. The higher the *IoU*, the better the image segmentation result. *DSC* ∈ (0, 1) is an evaluation matrix often used to evaluate the similarity between the ground truth and the segmentation results in medical image segmentation. The higher the *DSC*, the better the image segmentation results. For *Sen*, *Sp* and *Pre* ∈ (0, 1), the closer they are to 1, the better the segmentation effect.

### Experiment results

This section shows the segmentation results on lymph node metastasis dataset. In this paper, U-Net is set as the basic reference network. Based on this, we first assess the performance of the proposed Fovea-Unet and compare it with other improved networks based on U-Net. For a fair comparison, we implement their network architectures and utilize the same data preparation methods. Table 2 compares the segmentation results of the U-Net, U-Net + + , Double U-Net, Triple U-Net, and Attention U-Net in terms of all metrics used in our experiments. Analysis of Table 2 shows that all the improved networks achieve performance improvement compared with the original U-Net. As shown in Table 2, the Fovea-Unet achieves the best performance on five evaluation metrics except for *Sp* score, reaching 79.38%, 88.51%, 92.82%, 96.80%, and 84.57% for *IoU*, *DSC*, *Sen*, *Sp*, and *Pre*, respectively. Compared with the basic U-Net, Fovea-Unet increases its *IoU*, *DSC*, *Sen*, *Sp*, and *Pre* by 12.94%, 8.67%, 7.53%, 2.12% and 9.53%, respectively. In addition, compared with other networks, the detailed and non-local contextual information aggregation capability of Fovea-Unet improves the accuracy, such as *IoU* and *DSC*. Attention U-Net, with the advantage of attention, produces *IoU* and *DSC* results of 78.22% and 87.78%, respectively, which are only lower than those of our network. Significantly, the parameter amount of the proposed Fovea-Unet is only 23.23 MB, which even lower than Attention U-Net by 152.5 MB.

**Table 2:** Comparison results of the proposed network with other networks based on U-Net

Networks	*IoU*	*DSC*	*Sen*	*Sp*	*Pre*	Params/MB
U-Net	0.6644	0.7984	0.8529	0.9468	0.7504	147.59
U-Net + +	0.7255	0.8409	0.8913	0.9585	0.7959	184.58
Double U-Net	0.7290	0.8432	0.9072	0.9671	0.7877	173.99
Triple U-Net	0.7602	0.8564	0.9046	0.9626	0.8131	369.39
Attention U-Net	0.7822	0.8778	0.9212	**0.9693**	0.8383	175.76
Fovea-Unet	**0.7938**	**0.8851**	**0.9282**	0.9680	**0.8457**	**23.26**

Bold font indicates the best value for each metric

**Fig. 2:**
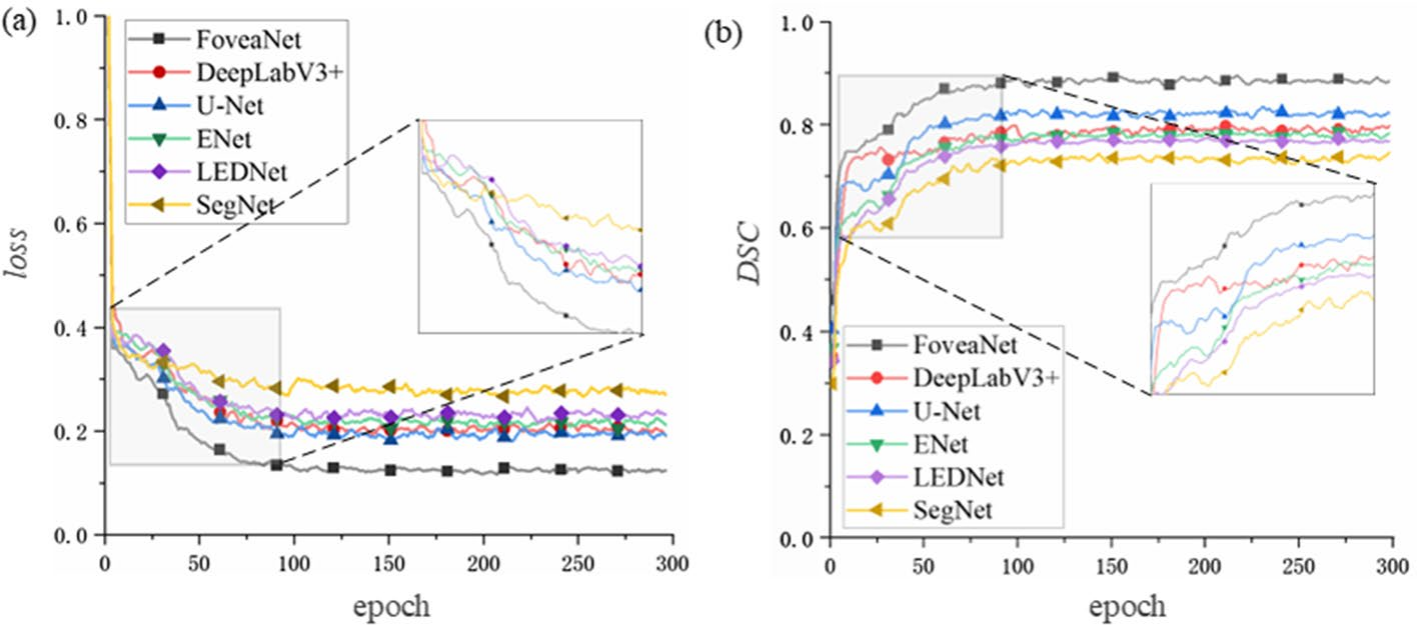
Training loss and *DSC* score of different networks with LNM dataset. **a**. Network training loss. **b** Network training *DSC*

To further verify the effectiveness and robustness of the Fovea-Unet proposed in this paper for lymph node metastasis segmentation, we selected some state-of-the-art segmentation networks for comparison, including three typical networks, namely, U-Net [5], SegNet [25], DeepLabv3 + [26], and two lightweight segmentation networks, namely, Enet [27], LEDNet [28]. The Fovea-Unet for LNM diagnosis performs well in the training process, as shown in Fig. 2. The training loss decrease rapidly to 0.15 after 100 epochs with Fovea-Unet, while other networks have similar trends but higher losses. The *DSC* increase simultaneously with the decrease of training loss. Among these networks, the Fovea-Unet achieves *DSC* of 88% after 125 training epochs, which is significantly better than other networks.

**Fig. 3:**
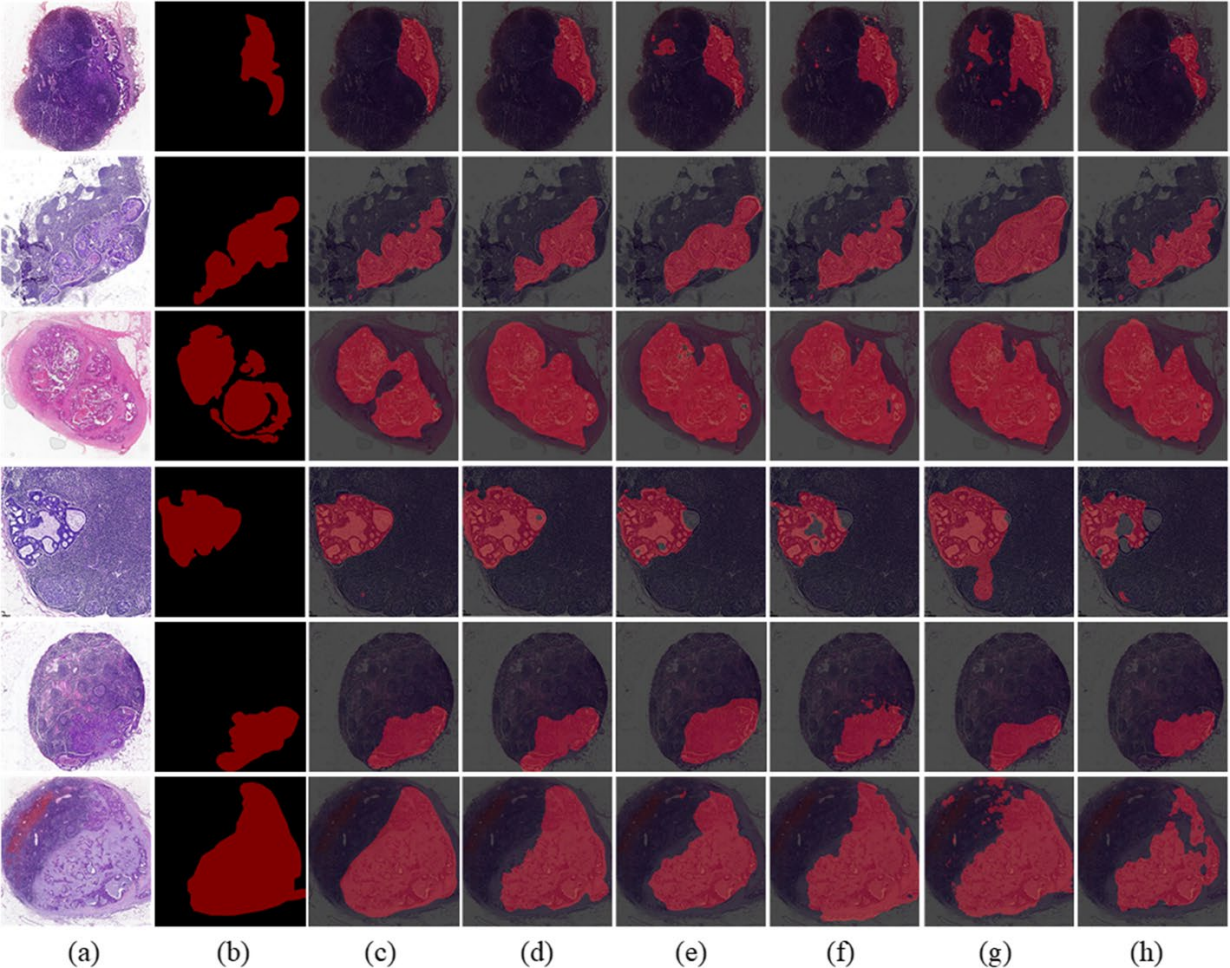
Segmentation results on LNM dataset of different networks. **a** original input images. **b** labels. **c** Fovea-UNet. **d** DeeplabV3 + . **e** U-Net. **f** ENet. **g** LEDNet. **h** SegNet

The performance comparison results are listed in Table 3. The Fovea-Unet performs significantly better than other networks for all metrics. Notably, in terms of metrics that have priority at the foreground pixel, it is observed that the proposed method shows superior performance with increments of 11.34%, 7.53%, 3.61% in *Sen*, 15.43%, 9.53%, and 8.06% in *Pre* compared with normal networks, respectively. Similarly, our proposed network shows great improvements of 13.61%, 6.48% in *Sen*, 9.87%, and 12.87% in *Pre,* respectively, while keeping the model parameters at the same level as two lightweight segmentation networks. Furthermore, Fig. 3 lists the segmentation results of different networks for several typical metastasis images on the lymph node metastasis data set and compare the corresponding segmentation prediction generated by overlaying segmentation masks on the input images. It is obvious that existing state-of-the-art networks under-segment regions with irregular shapes and low contrast characteristics, while Fovea-Unet performs extremely well (Rows 1–3).

**Table 3:** Comparison results of the proposed network with other state-of-the-art segmentation networks

Networks	*IoU*	*DSC*	*Sen*	*Sp*	*Pre*	Params/MB
SegNet	0.5975	0.7480	0.8148	0.9317	0.6914	112.32
U-Net	0.6644	0.7984	0.8529	0.9468	0.7504	147.59
DeepLabv3 +	0.7002	0.8237	0.8921	0.9491	0.7651	134.83
LEDNet	0.6245	0.7689	0.7921	0.9496	0.7470	8.65
Enet	0.6447	0.7840	0.8634	0.9382	0.7179	13.36
Fovea-Unet	**0.7938**	**0.8851**	**0.9282**	**0.9680**	**0.8457**	**23.26**

Bold font indicates the best value for each metric

## Discussion

The proposed Fovea-Unet improves the effectiveness and efficiency due to its several advantages:

1. The importance-aware Fovea Pooling (FP) is proposed to aggregate the detailed information and non-local contextual information which has ability to focus on what the region most relevant to LNM.
2. The improved H-GhostNet is proposed as a lightweight backbone network, promoting the ability of discriminative and heterogeneous feature extraction, improving the computation speed.

### Impact of different pooling strategies in FP

To discuss the effectiveness of the FP in Fovea-Unet, we conduct comprehensive ablation studies in terms of the aggregation method of the pooling layer and the boundary of pooling radius.

#### Effect of different pooling aggregation methods

The proposed Fovea-Unet respectively designs four FP in four stage of the encoder sub-network to refine and aggregate the information. To justify the effectiveness of FP, we first compare the results obtained when the FP is removed and the different pooling methods are employed including Lp Pooling [29], Average Pooling, and Mixed Pooling [30]. It should be noted that the Max Pooling was not used as a comparative method, because the characteristic of only selecting the largest element will harm the network segmentation performance.

As detailed in Table 4, the three different pooling methods in the FP greatly improve the segmentation performance with the baseline denoted as identity map, where the IM denotes the identity map, LP denotes the Lp Pooling method, AP denotes the Average Pooling, and MP denotes the Mixed Pooling. For the evaluation metrics on four different methods, the *IoU* increase by 12.95%, 11.70%, and 12.92%, respectively, and *DSC* increase by 8.66%, 7.89% and 8.66%, respectively, which signifies the effectiveness of FP for segmentation tasks. Segmentation metrics show that three methods all achieve good performance and have an average score of 79.98% and 88.25% in terms of *IoU* and *DSC*. Moreover, Mix Pooling and Lp Pooling get a relatively higher score than the Average Pooling, indicating that appropriate proportion of maximum information is important for good segmentation performance on LNM region. From the results presented in Table 4, it is obvious that the great improvement is brought by FP with low correlation to the chosen pooling methods in the FP.

**Table 4:** Comparison results of the proposed network under different pooling aggregation methods

Methods	*IoU*	*DSC*	*Sen*	*Pre*	Params/MB
IM	0.6646	0.7985	0.8531	0.7505	11.98
LP	0.7941	0.8852	0.9280	0.8462	23.26
AP	0.7816	0.8774	0.9140	0.8437	23.26
MP	0.7938	0.8850	0.9282	0.8457	23.26

#### Effect of different pooling boundaries

**Fig. 4:**
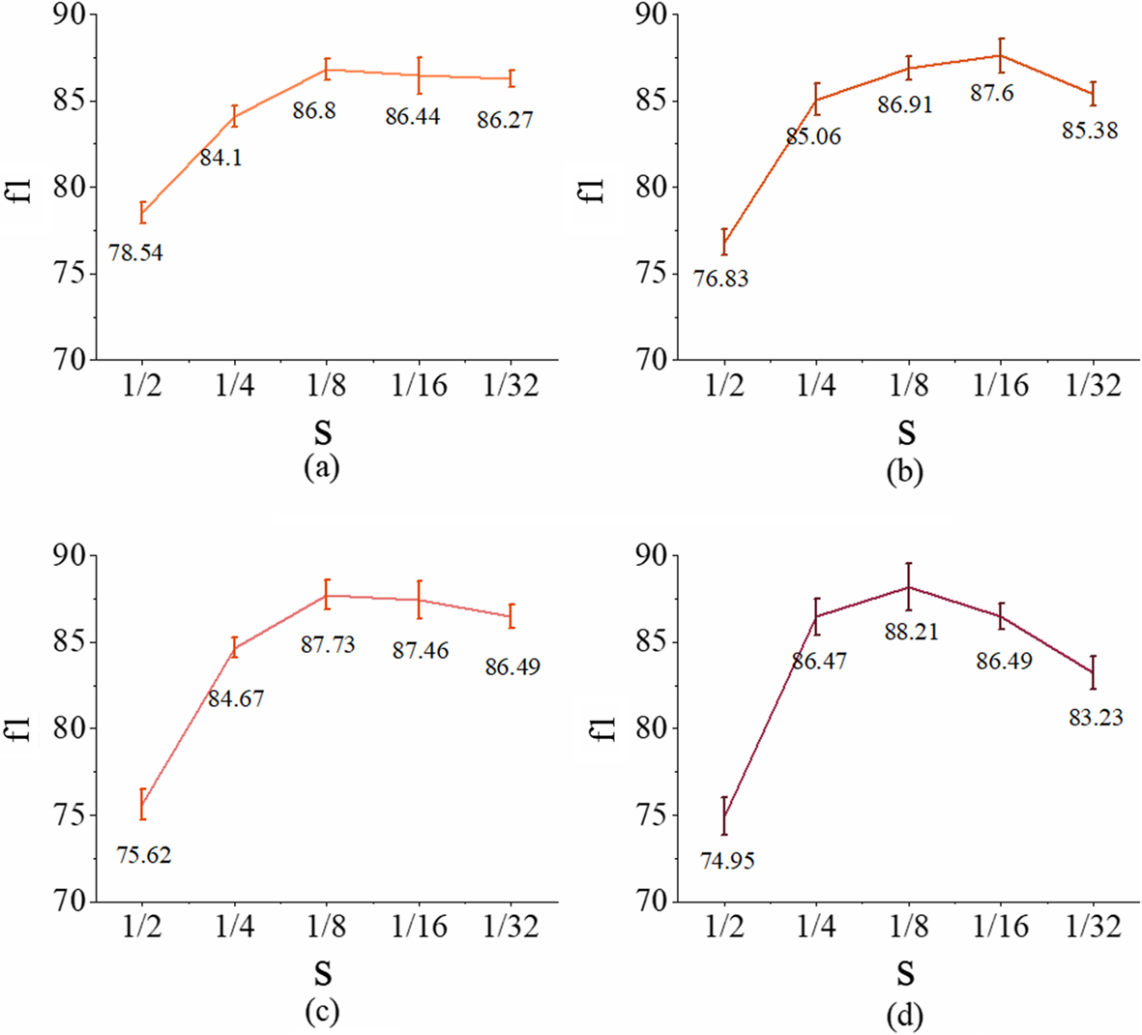
Performance of different pooling boundaries in each stage. **a** stage 1. **b** stage 2. **c** stage 3 **d** stage 4

When the importance of a specific element $z_{k}^{i}$ is set to zero, the pooling radius reaches its maximum, i.e., $r = e^{\varsigma }$. $\varsigma$ is an empirical value associated with the maximum pooling radius, namely, pooling boundaries [see the Eq. (6) in “Methods” section for details]. In this section, we discuss the impact of different pooling boundaries on the segmentation performance and how to set the value of pooling boundaries in a comparative experiment conducting with five different scales in each stage of encoder sub-network. It is worth noting that we use a normalization term $s = e^{\varsigma } /w_{i}$ to denote the pooling boundary, where $w_{i}$ represent the spatial size of feature $i$. For the output features of the four stages in the encoder, the parameters $s$ is first set to 1/8, and then the parameters are adjusted stage by stage until the best results are obtained. In each stage, five different experiments of pooling boundaries from 1/2 to 1/32 are conducted, which is shown in Fig. 4.

From the comparative results, the Fovea-Unet gets the best performance when $s = 1/16$ or $s = 1/8$, while suboptimal performance is got with reduction of pooling boundary and sharp decline of performance is shown with increasingly pooling boundary. In the deeper stage of 3 and 4, the trend of peak value is strengthened in both extreme, at the same time, variance of performance is also nearly doubled compared to the stage 1 and stage 2. The main reasons are as follows. In the low-level feature maps, each location represents small local neighborhood information and the shallow features take a majority of images information, which is responsible for detailed contextual information but making decision in a small extent. In contrast, with the increased receptive filed gradually, each element of the high-level feature maps has larger non-local perception and semantic information that contributes to the segmentation results in a greater extent. Hence, in different stages, it is more advantageous to employ a proper combination of pooling boundaries to explore both the detailed information and non-local contextual information for a better guidance of the FP, so as to improve the performance of segmentation network. The optimal value of $s$ in each stage should be set according to Fig. 4, i.e., $s = 1/8$ in stage 1, $s = 1/16$ in stage 2, $s = 1/8$ in stage 3, and $s = 1/8$ in stage 4.

### Impact of different backbones in Fovea-Unet

We also compare the proposed backbone H-GhostNet with other backbones. Moreover, we demonstrate the effectiveness of HSIC regularization.

#### Quantitative analysis of different backbones

To investigate the effectiveness of different backbones to the proposed method, we compare the proposed H-GhostNet with five different state-of-the-art backbones, which include three normal backbones, namely, VGGNet [31], ResNet [32], InceptionNet [33], and two lightweight backbones, namely, MobileNet [34], GhostNet [23]. All of them can extract the abundant feature information at the shallow level and provide the discriminative feature at the high level. For a fair comparison, we also implement their network architectures and utilize the same parameter initialization methods. Our proposed H-GhostNet considers the heterogeneous feature generation as a regularization term of the loss function. In Table 5, we report the results of the proposed method with different backbones, from which we can see that the proposed H-GhostNet performs favorably against other backbones. It is obvious that the Fovea-Unet with the backbone of H-GhostNet achieves prominent performance with *IoU* 79.38%, *DSC* 88.51%, *Sen* 92.82%, and *Pre* 84.57%. In particular, our method shows the increments of 2.73%, 0.17%, 1.65%, 2.35%, 2.27%, and 0.99% in terms of comprehensive metric of *DSC* compared to above backbones. We observe that ResNet as the backbone has a faint superiority, probably due to the deeper network architecture with 50 layers. Nonetheless, the amount of space tied up by ResNet would make the network bloated. Among the performance with lightweight backbones, the accuracy metrics are declining in varying degrees with the decrease of model parameter quantity. In addition, it is worth noting that the proposed H-GhostNet significantly improves the segmentation accuracy of the baseline backbone, GhostNet, and achieves improvements of 1.57% in *IoU*, 0.99% in *DSC*, 1.84% in *Sen*, and 0.26% in *Pre*, which validate the regularization of the intrinsic feature-based topology. Overall, these comparable accuracy results reveal the good capability of H-GhostNet to effectively extract features from the training dataset while keeping the smallest memory occupation compared to both normal and lightweight backbones.

**Table 5:** Comparison results of the proposed network under different backbones

Backbones	*IoU*	*DSC*	*Sen*	*Pre*	Params/MB
VGG	0.7511	0.8578	0.9026	0.8173	103.05
ResNet	0.7911	0.8834	0.9250	0.8453	241.68
Inception	0.7678	0.8686	0.9127	0.8286	75.12
MobileNet	0.7569	0.8616	0.8998	0.8266	48.83
GhostNet	0.7781	0.8752	0.9098	0.8431	23.26
H-GhostNet	**0.7938**	**0.8851**	**0.9282**	**0.8457**	**23.26**

Bold font indicates the best value for each metric

#### Effectiveness of HSIC regularization

**Fig. 5:**
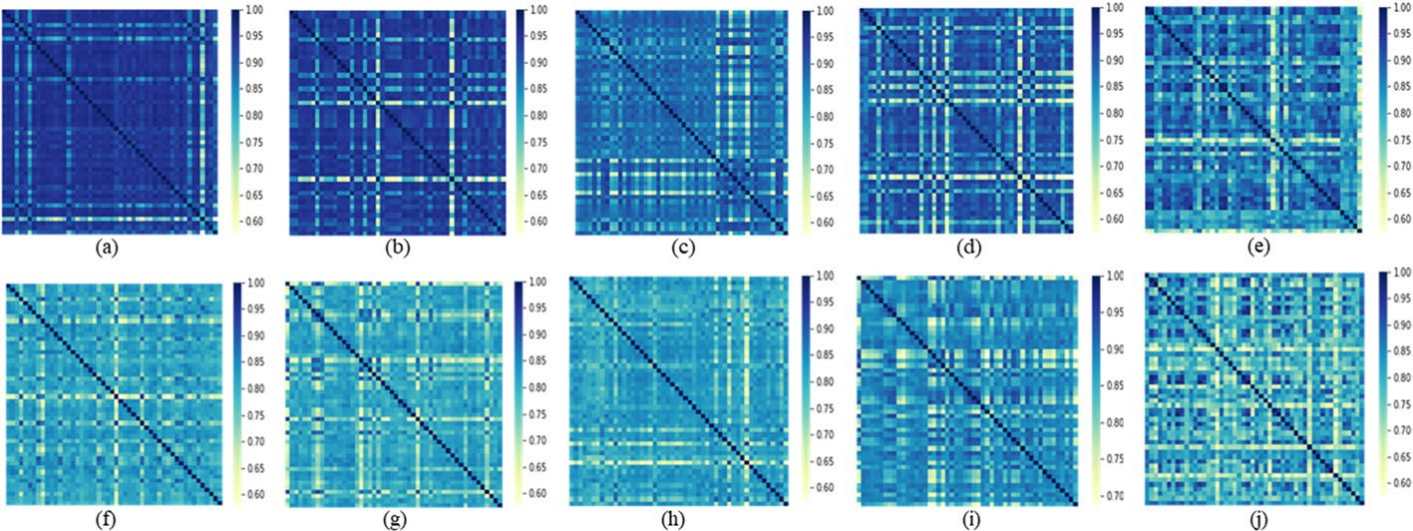
CKA similarity heatmap of GhostNet backbone among the first fifty channels of intrinsic features for two cases, including without LHSIC **a**–**e** and with LHSIC **f**–**j**. **a**, **f** layer 8. **b**, **g** layer 10. **c**, **h** layer 12. **d**, **i** layer 14. **e**, **j** layer 16

The effect of HSIC regularization is further explored through the visualization of channelwise feature similarity. We continue our investigation using CKA to study the internal representation structure of specific layers, which enable quantitative comparisons of features within networks [35]. As shown in Fig. 5, the first 50 intrinsic feature maps within a specific layer are taken as the input to generate a heatmap with the x and the y axes indexing ordered representations. Darker color represents the higher similarity when the Fovea-Unet is trained without the regularization, it is observed that intrinsic features extracted by a specific layer have different statistic properties with different training strategies. In Fig. 5a–e, features extracted without regularization tend to be homogenous, we visualize the same situation except for the extra similarity regularization $L_{HSIC}$ in Fig. 5f–j. It results in relatively low channelwise similarity, which confirms that the H-GhostNet regularized by the similarity constraint can effectively promote the capability of Fovea-Unet. In the future, the devised H-GhostNet can be utilized to facilitate the medical segmentation tasks with the complementary knowledge of features.

### Limitation and future work

Although promising results have been obtained, there still are some limitations in the proposed Fovea-Unet that should be taken into consideration. On one hand, the attention-based importance-aware modules would result in large number of floating-point operations per second (FLOPs) with high computational costs and the calculation process of pooling radius is relatively tedious. On the other hand, the single-head FP would hard to cope with the situations of extremely scattered metastasis. In the future work, more efficient computing methods can be used in the importance-aware modules, and the multi-head FP can be developed with reference to the multi-head attention mechanism in Transformer, which makes the segmentation network more flexible in feature aggregation and further improves the quality of LNM segmentation.

## Conclusion

Automatic diagnosis of lymph node metastasis on colorectal cancer is challenging due to the dilemma of aggregating the detailed information and non-local contextual information. In this paper, we propose a novel importance-aware FP to tackle the aforementioned issue. The FP adopts an importance-aware module and a pooling layer with adaptive radius to adjust the resolution of different regions to aggregate detailed and non-local contextual information, so that the network can focus on the LNM region with high diagnostic value. On this basis, an improved lightweight backbone H-GhostNet is developed for reduce the computational burden of FP on the entire network. H-GhostNet utilizes the feature-based similarity regularization to enhance the ability of discriminative and heterogenous feature extraction. Based on the quantitative and qualitative analysis of segmentation results, it can be concluded that our method outperformed all other methods based on deep learning by a large margin while keeping a low model parameter cost. The comprehensive experiments demonstrate the superiority of the proposed methods, which inherently can be transferred to extensive medical image segmentation baseline for powerful feature extraction and aggregation ability.

## Methods

### Overall network architecture

**Fig. 6:**
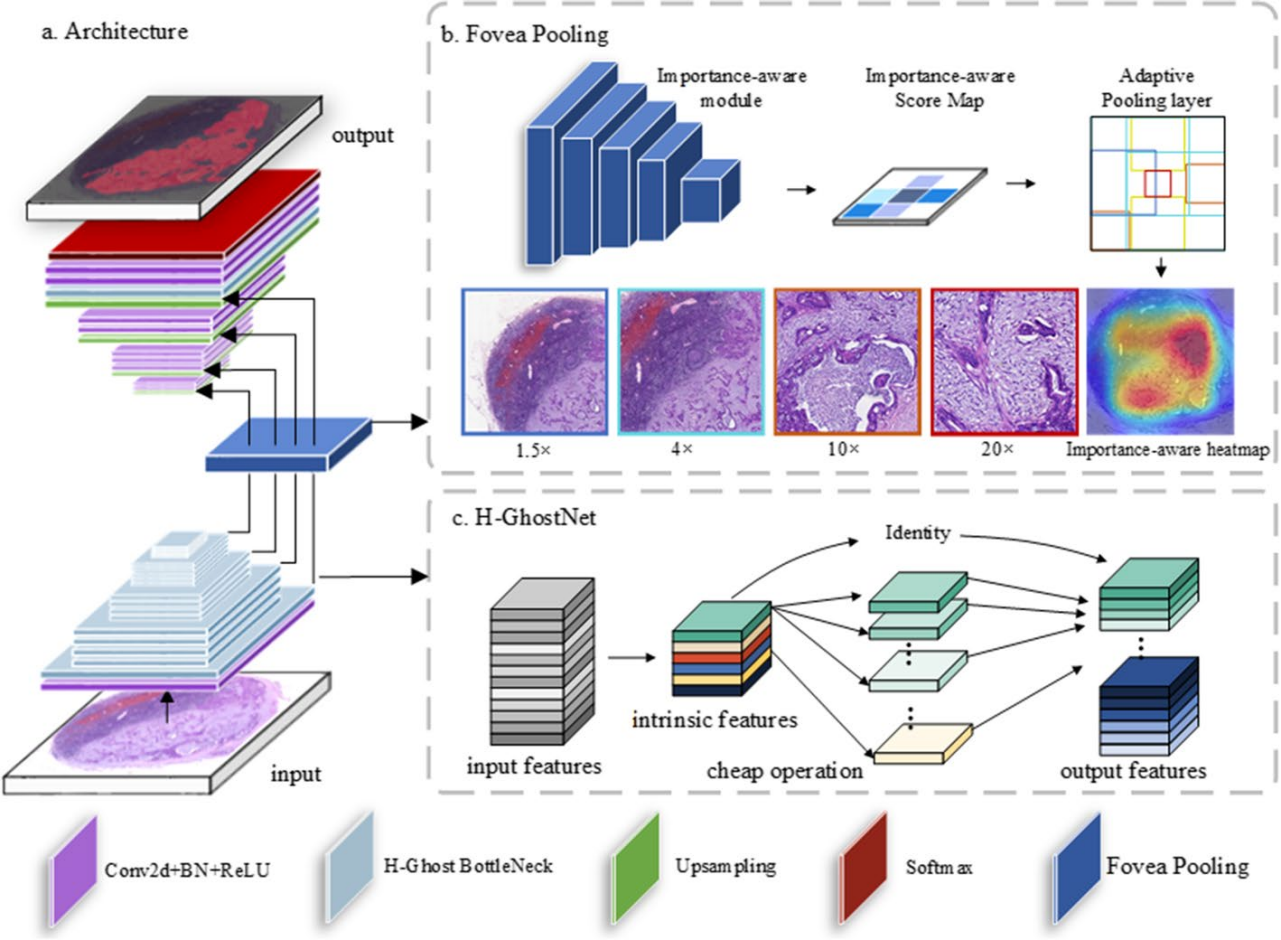
The overall structure of the proposed Fovea-UNet. **a**. The architecture of Fovea-UNet. Medical input images are first fed into the extracting path and four intermediate features maps are obtained. Then the Fovea Pooling modules take the feature maps as input and yield the output respectively. Lastly, the segmentation mask is acquired by concatenating the output of FP in turn and upsampling layers hierarchically. **b**. The illustration of Fovea Pooling. The importance-aware module calculates the importance-aware map using the intermediate features as input, and the importance-aware map of each feature provides the basis of the pooling radius. We map the pooling process on the original input images as the illustration, which is shown in the upper part of (**b**). The closer to warm the color of the picture border is, the more the picture contains detailed information. **c**. The illustration of the HSIC-Ghost convolution layer. The constraint of the normal convolution layer that generates the distinct intrinsic features is added and then we adopt more cheap operations to ensure the distinction and sufficiency of features

As shown in Fig. 6, the proposed Fovea-Unet is built and extended on the U-Net architecture, which mainly consists of a CNN encoder for extracting image features from different layers and a CNN decoder for pixelwise segmentation. In the encoder sub-network, to produce richer contextual information and aggregate them in a better manner, we replace the identity map that lay in the skip connection with four FP modules. The importance-aware modules of FP first take the intermediate feature maps Fi, $i \in \{ 1,2,3,4\}$ as input, and yields the importance-aware map Pi, $i \in \{ 1,2,3,4\}$, respectively. Then, the pooling layer is adopted to aggregate the feature in the spatial domain, where the pooling radius depends on the importance-aware of specific feature map and the pooling step is one. Meanwhile, the H-GhostNet is adopted as the backbone network for feature extraction, where the last global pooling and fully connected layers of H-GhostNet are removed. Only one convolution and four bottlenecks for primary feature extraction are retained, where each bottleneck contains four H-Ghost convolution layers. Without loss of generality, for an input image, the output features of four bottlenecks are Fi, $i \in \{ 1,2,3,4\}$ mentioned above. The output size of each feature is 1/2, 1/4, 1/8, and 1/8 of the input image. Once this encoding process is finished, the encoding features are concatenated with the decoder output in turn for the final generation of the segmentation mask.

### Fovea pooling

Information aggregation is great importance for segmentation network in capturing detail and no-local contextual information [36]. General information aggregation is modeled as:

2 $${\mathbf{z}}_{i} = \frac{1}{N}\sum\limits_{\forall j \in \Omega (i)} {F({\mathbf{x}}_{i} ,{\mathbf{x}}_{j} ,\Delta_{ij} ){\mathbf{x}}_{j} }$$

where ${\mathbf{z}}_{i}$ is the newly aggregated feature at the position $i$, and ${\mathbf{x}}_{i}$ is the feature at position $i$ in the input feature map ${\mathbf{X}}$. $\forall j \in \Omega (i)$ enumerates all positions in the region of interest associated with $i$, and $\Delta_{ij}$ represents the relative location of position $i$ and $j$. $F({\mathbf{x}}_{i} ,{\mathbf{x}}_{j} ,\Delta_{ij} )$ can be any function or learned parameters according to the operation and it represents the information flow from $j$ to $i$. Note that taking relative location $\Delta_{ij}$ into account $F({\mathbf{x}}_{i} ,{\mathbf{x}}_{j} ,\Delta_{ij} )$ is sensitive to different relative locations. In addition, $N$ is for normalization. Although these attention methods successfully capture the importance and relationship between different areas from the perspective of information flow, they ignore the further highlight of the most important area that contributes to segmentation results. Thus, the essence of features could not be fully revealed which downgrades the segmentation accuracy.

To remedy these drawbacks, the Fovea Pooling inspired by human retinal Fovea is proposed to dynamically aggregate the detailed information of important areas and non-local contextual information of other areas based on the capacity for adaptively adjust the pooling radius according to the importance-aware of information. The proposed Fovea Pooling consists of an importance-aware module and the pooling layer with adaptive radius. First, the pixel-level importance of features is calculated through the importance-aware module evolved from PSANet [37]. Then, the pooling layer aggregates the features with variable pooling radius which has an inverse trend of the pixel-level importance.

**Fig. 7:**
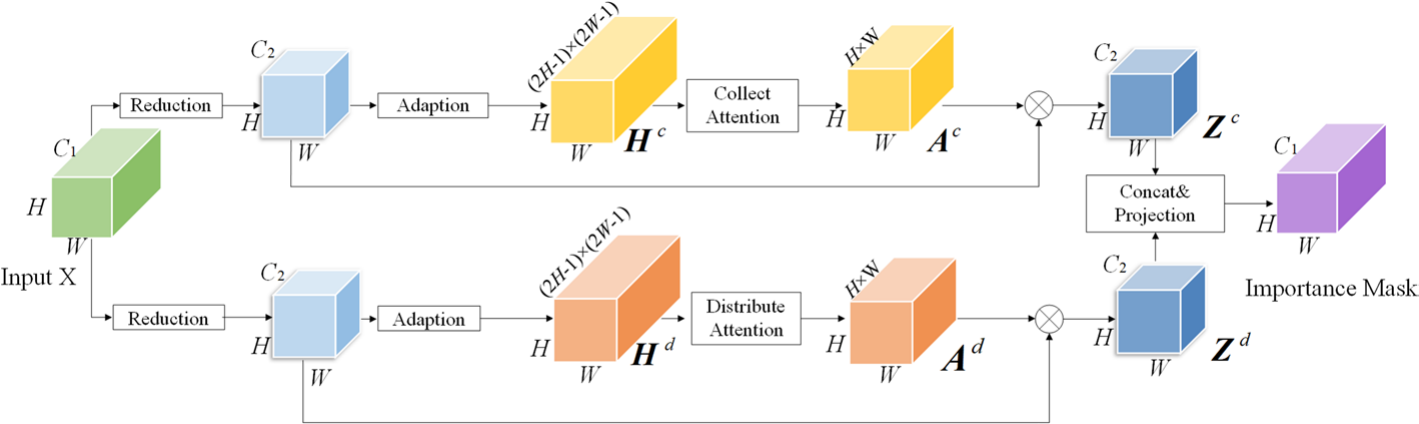
Architecture of importance-aware module

Specifically, for the importance-aware module, the architecture follows the PSA module of PSANet in general. PSA module as a pointwise spatial attention module, aiming to adaptively obtain the information over the entire feature map, provides an implementation method to get the pixel-level importance of features for this work. Compared with PSA module, the importance-aware module only remains the architecture of generating pixelwise global attention maps for each position in feature map **X** through several convolutional layers as the importance-aware module in our Fovea Pooling. The specific architecture of the importance-aware module followed PSANet is shown in Fig. 7.

As illustrated in Fig. 7, the importance-aware module adaptively predicts two global importance-aware maps ${\mathbf{Z}}$ for each position in the feature map ${\mathbf{X}}$ by two parallel branches, i.e., collect branch and distribute branch. In the collect branch, at each position $i$, we predict how current position is related to other positions based on feature at position $i$. In addition, vice versa, the distribute branch is used to distribute the information at the current position to assist the prediction of other ones. Hence, Eq. (2) is rewritten as:

3 $${\mathbf{z}}_{i} \, = \,\frac{1}{N}\,\sum\limits_{\forall j} {a_{i,j}^{c} \,{\mathbf{x}}_{j} } \, + \,\frac{1}{N}\,\sum\limits_{\forall j} {a_{i,j}^{d} \,{\mathbf{x}}_{j} }$$

where $a_{i,j}^{c}$ and $a_{i,j}^{d}$ denote the predicted attention values in the pointwise attention map ${\mathbf{A}}^{c}$ and ${\mathbf{A}}^{d}$ from collect and distribute branches, respectively. Before this, the intermediate attention maps ${\mathbf{H}}^{c}$ and ${\mathbf{H}}^{d}$ is calculated as the over-completed map both with the spatial size of $H \times W$ and $(2H - 1) \times (2W - 1)$ channels. According to this, the element at $s_{th}$ row and $t_{th}$ column in the attention mask $a_{{\left[ {k,l} \right]}}^{c}$ is:

4 $$a_{[k,l]}^{c} = {\mathbf{h}}_{[k,l],[H - k + s,W - l + t]}^{c} , \, \forall s \in {[0},H{), }t \in [0,W)$$

where $[ \cdot , \, \cdot ]$ indexes position in rows and columns, and ${\mathbf{h}}^{c}$ indicates the reshaped feature embedding at the position $[k,l]$ with size of $(2H - 1) \times (2W - 1)$. Similar to the collect branch, the element of distribute attention mask $a_{[k,l]}^{d}$ is computed as:

5 $$a_{[k,l]}^{d} = {\mathbf{h}}_{[k,l],[H - k + s,W - l + t]}^{d} , \, \forall s \in {[0},H{), }t \in [0,W)$$

These two maps $a_{{\left[ {k,l} \right]}}^{c}$ and $a_{[k,l]}^{d}$ encode the context dependency between different position pairs in a complementary way, leading to improved information propagation and enhanced utilization of long-range context.

In the pooling layer, global importance-aware map ${\mathbf{Z}}$ is regarded as calculation basis and pooling radius $r_{k}$ in each position is decided by the corresponding importance:

6 $$r_{k} = \left\lfloor {e^{{(\varsigma \cdot (1 - {\mathbf{Z}}_{k}^{i} ))}} } \right\rfloor$$

where $r_{k}$ denotes the pooling radius in position $k$, ${\mathbf{Z}}_{k}^{i}$ denote the importance in the position $k$ of the layer $i$, and $\varsigma$ is an empirical value. We take this empirical equation that make the region with high importance maintain high resolution and $r_{k} = 1$ if ${\text{Z}}_{k}^{i} = 1$, while the other extreme is $r_{k} = \left\lfloor {e^{\varsigma } } \right\rfloor$ if ${\text{Z}}_{k}^{i} = 0$. It ensures the radius decline rapidly along with the linear increase of normalized importance, which variable factors can be synthetically considered and used to the utmost limits for reaching the optimum effect on feature aggregation.

Without the loss of generality, we take the general aggregation method of pooling layer as the example for the illustration of the backpropagation process. The output of pooling layer ${\mathbf{W}}$ is:

7 $${\mathbf{W}}_{i}^{N} = \frac{1}{n}\sum\limits_{{\forall j \in \Omega_{i} }} {\eta_{j} {\mathbf{W}}_{j}^{N - 1} }$$

where ${\mathbf{W}}_{i}^{N}$ and ${\mathbf{W}}_{j}^{N - 1}$ indicate the feature element of layer $N$ and layer $N - 1$, respectively, and $j$ was employed to point the all position of pooling window at the position $i$. $\eta_{j}$ denotes the weight of feature in the specific position. In the backpropagation stage, the gradient of relative element is calculated as:

8 $$\frac{{\partial W_{i}^{N} }}{{\partial W_{j}^{N - 1} }} = \frac{1}{n}\eta_{j}$$

According to Eq. (8), both the weight of feature $\eta_{j}$ and the number of elements $n$ in the receptive field together have determined gradients of training samples. There are only a few elements in the high importance region, so the backpropagation process will give the feature elements in this region a larger gradient, that is, the more important the region will maintain a higher resolution, so that the feature elements in the region will get more attention. Therefore, FP has the capacity to extract robust and discriminative features through stochastic gradient descent (SGD) in the semantic segmentation network. In this way, FP can effectively aggregate the pixel-level semantic information and dynamically control the receptive field size, so that the input features that directly contribute to the segmentation result remain high resolution, while the no-local contextual information is responsible for by the large receptive field region.

### H-Ghost backbone

GhostNet is an impressive alternative backbone designed to decrease computational costs of the generic convolutional layer while preserving the similar ability of feature extraction to original convolutional layer. The key assumption of the GhostNet is embracing feature redundancy and generating redundancy through the cheaper linear operation on the intrinsic feature maps. In practice, given the input data ${\varvec{X}} \in {\mathbf{R}}^{{{\text{c}} \times h \times w}}$, where $c$ is the number of channels and $h$ and $w$ are the height and width of the input data, respectively. The operation of the primary convolution layers for producing *m* intrinsic feature maps ${\varvec{I}} \in {\mathbf{R}}^{{m \times h{\prime} \times w{\prime} }}$ can be formulated as ${\varvec{I}} = {\varvec{X}} * f + {\varvec{b}}$, where $*$ is the convolution operation, ${\varvec{b}}$ is the bias term, $f \in {\mathbf{R}}^{c \times k \times k \times m}$ is the convolution filters in a specific layer and $k \times k$ is the kernel size of $f$. To further increase the feature redundancy, a series of fast linear transformations on each intrinsic feature ${\varvec{I}}_{i}$ is performed:

9 $${\varvec{y}}_{ij} = \Phi_{i,j} \left( {{\varvec{I}}_{i} } \right), \, \forall i = 1, \ldots m, \, j{ = 1,} \ldots ,s$$

where ${\varvec{I}}_{i}$ is the $i$ th intrinsic feature map in ***I***, and the $\Phi_{i,j}$ is the $j$ th linear transformation for generating the $j$ th ghost feature map ${\varvec{y}}_{ij}$. However, if GhostNet is directly used as the backbone, although it can generate feature maps with redundant features through cheap linear operations, it is not suitable as an encoder for segmentation networks directly. On one hand, in the case of the complex, variable LNM to be segmented, the limited number of intrinsic features can’t guarantee the full mining of semantic information. On the other hand, the process of generating intrinsic features in GhostNet only uses normal convolution layers, which can’t ensure the heterogeneity among features, and seriously affects the segmentation results of the entire network. Hence, learning the sufficient and redundant intrinsic feature representations more efficiently will be beneficial. Information theory underlying much research on deep learning as well as neuroscience offers an effective way to address this issue. HSIC is the Hilbert–Schmidt norm of the cross-variance operator between the distribution in Reproducing Kernel Hilbert Space (RKHS), which is widely used as a dependency measurement of representations in the deep learning literature [24]. The formulation of HSIC is:

10 $$\begin{gathered} HSIC({\rm P}_{XY} ,H,G) = \left\| {C_{XY} } \right\| \\ = E_{{XYX^{\prime}\,Y^{\prime}}} [k_{X} (X,X^{\prime})k_{{Y^{\prime}}} (Y,Y^{\prime})] \\ + E_{{XX^{\prime}}} [k_{X} (X,X^{\prime})]E_{{Y^{\prime}}} [k_{{Y^{\prime}}} (Y,Y^{\prime})] \\ - 2E_{XY} [E_{{X^{\prime}}} [k_{X} (X,X^{\prime})]E_{{Y^{\prime}}} [k_{{Y^{\prime}}} (Y,Y^{\prime})] \\ \end{gathered}$$

where *k*_*X*_ and *k*_*Y*_ are kernel functions. ***H*** and ***G*** are the Hilbert spaces, and *E*_*XY*_ is the expectation over *X* and *Y*.

In the above intuition, we incorporate the normalized HSIC and proposed H-GhostNet to learn the discriminative and complementary representations, which made the original GhostNet more efficient and unchallenged by adding a regularization term of HSIC. It imposes the orthogonal constraint on learned intrinsic features and leaves room for more redundancy in the cheap operation. Let $D: = \left\{ {(x_{1} ,y_{1} ), \ldots (x_{m} ,y_{m} )} \right\}$ denotes *m* independently identical distribution samples draw from ${\text{P}}_{XY}$, where ${\varvec{x}}_{i} \in {\mathbf{R}}^{{d_{x} }}$ and ${\varvec{y}}_{i} \in {\mathbf{R}}^{{d_{y} }}$. Then, Eq. (10) leads to the following empirical expression:

11 $${\text{HSIC}}\,(D,H,G)\,=\,(m\, - \,1)^{ - 2} \,tr({\mathbf{K}}_{X} {\mathbf{HK}}_{Y} {\mathbf{H}})$$

where ${\mathbf{K}}_{X} \in {\mathbf{R}}^{m \times m}$ and ${\mathbf{K}}_{Y} \in {\mathbf{R}}^{m \times m}$ both have entries ${\mathbf{K}}_{Xij} = k({\mathbf{x}}_{i} ,{\mathbf{x}}_{j} )$ and ${\mathbf{K}}_{Yij} = k({\mathbf{y}}_{i} ,{\mathbf{y}}_{j} )$, and ${\mathbf{H}} \in {\mathbf{R}}^{m \times m}$ is the centering matrix ${\mathbf{H}} = {\mathbf{I}}_{m} - \frac{1}{m}{\mathbf{1}}_{m} {\mathbf{1}}_{m}^{T}$. In this paper, we devise a loss function $L_{nHSIC}$:

12 $$L_{nHSIC} \, = \,\alpha \,\sum\limits_{\forall i,j \in [1,m], \, i \ne j} {nHSIC\,({\mathbf{I}}_{i} ,\,{\mathbf{I}}_{j} )}$$

Where ${\mathbf{I}}_{i} \, \in \,{\text{R}}^{s\, \times \,p}$ is the representation within the intrinsic feature maps, with $p$ neurons, evaluated on the same s samples. We use the normalized-HSIC (nHSIC) that is the normalized Hilbert–Schmidt independence criterion based on the normalized cross-covariance operator, given by

13 $${\text{nHSIC}}\, = \,{\text{tr}}\,(\widetilde{{\mathbf{K}}}_{{I_{i} }} \,\widetilde{{\mathbf{K}}}_{{I_{j} }} )$$

where $\widetilde{{\varvec{K}}}_{{I_{i} }} = \overline{{\varvec{K}}}_{{I_{i} }} (\overline{{\varvec{K}}}_{{I_{i} }} + \varepsilon m{\mathbf{I}}_{m} )^{ - 1}$ and $\widetilde{{\varvec{K}}}_{{I_{j} }} = \overline{{\varvec{K}}}_{{I_{j} }} (\overline{{\varvec{K}}}_{{I_{j} }} + \varepsilon m{\mathbf{I}}_{m} )^{ - 1}$. $\overline{{\varvec{K}}}_{{I_{i} }}$ and $\overline{{\varvec{K}}}_{{I_{j} }}$ denote centered kernel matrices, and $\varepsilon$ is a small constant. In this way, the proposed H-GhostNet can extract the comprehensive and distinct intrinsic feature representations towards LNM diagnosis while reducing the computational burden.

### Loss function

In the actual cancer segmentation task, there is a highly imbalance distribution between the tumor and non-tumor regions, which leads to the poor performance of segmentation network. Therefore, a suitable loss function is crucial to alleviate the above problem.

Focal loss [38] is taken to alleviated the problem, along with the similarity loss:

14 $$L = L_{focal} + \lambda L_{nHSIC}$$

where $\lambda$ is empirically set to 0.75. The Focal loss function is computed as follows:

15 $$L_{focal} = - \alpha_{t} (1 - p_{t} )^{\gamma } \log (p_{t} )$$

where $p_{t} = p$ if $y = 1$, $p_{t} = 1 - p$ if $y = 0$. $a_{t}$ is used to restrain the imbalance between the number of positive and negative samples, and $\gamma$ to control the imbalance of hard or easy samples.

## Data Availability

Availability of data and materials. The data sets generated and/or analyzed during the current study are not publicly available due to hospital information protection mechanism, but are available from the corresponding author on reasonable request.

## Abbreviations

LNM: Lymph node metastasis
WSI: Whole slide image
FP: Fovea pooling
CRC: Colorectal cancer
CNNs: Convolutional neural networks
HSIC: Hilbert–Schmidt independence criterion
nHSIC: Normalized-HSIC
HE: Hematoxylin–eosin
FLOPs: Floating-point operations per second
SGD: Stochastic gradient descent
RKHS: Reproducing Kernel Hilbert Space
